# Controlling blood pressure soon after intracerebral hemorrhage: The SAMURAI-ICH Study and its successors

**DOI:** 10.1038/s41440-022-00866-8

**Published:** 2022-03-08

**Authors:** Kazunori Toyoda, Masatoshi Koga, as the SAMURAI Investigators

**Affiliations:** Department of Cerebrovascular Medicine, National Cerebral and Cardiovascular Center, Suita, Japan

**Keywords:** Aacute stroke, Antihypertensive therapy, Hemorrhagic stroke, Hypertension, Nicardipine

## Abstract

The impact of acute therapy for intracerebral hemorrhage (ICH) lags far behind that for acute ischemic stroke. Intensive blood pressure lowering is a promising therapeutic strategy for acute ICH, especially for East Asian patients whose etiological mechanism is more commonly hypertension than that of patients in the Western population. A multicenter, prospective, observational study named the Stroke Acute Management with Urgent Risk-factor Assessment and Improvement-IntraCerebral Hemorrhage (SAMURAI-ICH) study, involving 211 patients from ten Japanese stroke centers, was performed to elucidate the safety and feasibility of blood pressure lowering to 160 mmHg or less in acute ICH patients using intravenous nicardipine. When we started the study, intravenous nicardipine was not officially approved for hyperacute ICH patients in Japan. The SAMURAI-ICH study was also a pilot study to judge the feasibility of participation by many Japanese investigators in an international, randomized, controlled trial named the Antihypertensive Treatment of Acute Cerebral Hemorrhage (ATACH)−2 trial. The SAMURAI-ICH study, ATACH-2 trial, and their combined individual participant data meta-analysis produced several new interesting findings on how to control blood pressure levels in acute ICH patients. Some of the findings are introduced in the present review article.

## Graphical abstract

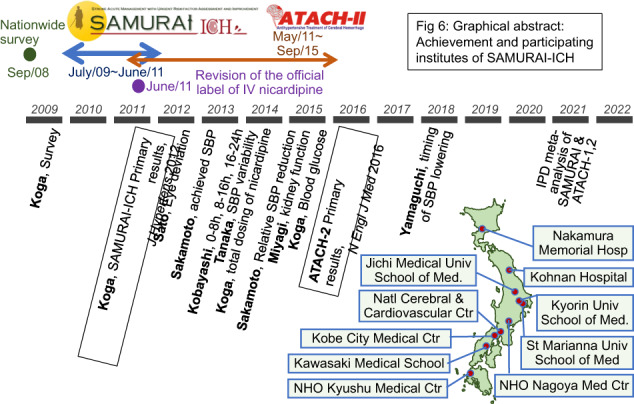

## Introduction: before the SAMURAI-ICH study

**Fig. 1:**
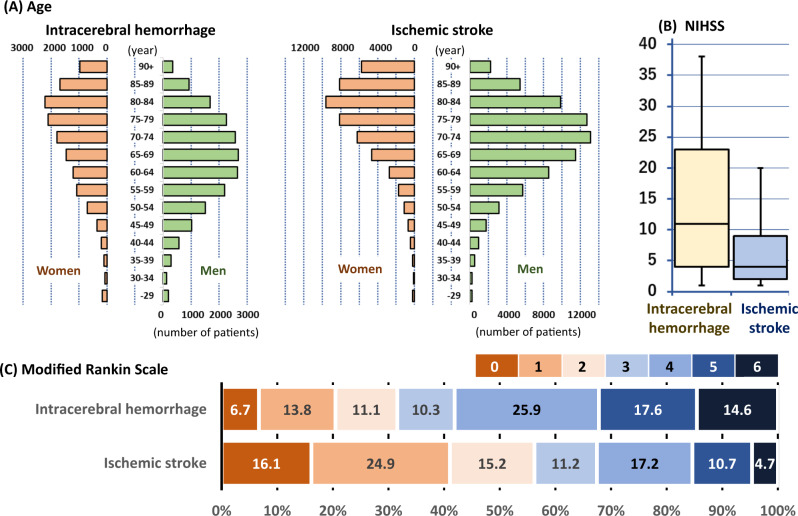
Age at onset (**A**), initial National Institutes of Health Stroke Scale scores (**B**), and discharge modified Rankin Scale scores (**C**) of 33,178 patients with acute intracerebral hemorrhage and 125,722 patients with acute ischemic stroke from a multicenter registry between 2000 and 2018: The Japan Stroke Data Bank. Edited based on the data from Ref [2]. The National Institutes of Health Stroke Scale (NIHSS), a serial measure of neurological deficit, is a 42-point scale that quantifies neurological deficits in 11 categories, with a score of 0 indicating normal function without neurological deficit and higher scores indicating a greater severity of deficit. The modified Rankin scale grades the degree of disability or dependence in daily activities using scores ranging from 0 (no symptoms) to 6 (death). In Panel (**B**), the boxes represent the interquartile ranges, the lines across the boxes indicate the median values, and the whiskers represent the 10th percentile and 90th percentile values

Hemorrhagic stroke, including intracerebral hemorrhage (ICH) and subarachnoid hemorrhage, is much more devastating than ischemic stroke. Although new cases of hemorrhagic stroke were estimated to account for 30% of the overall new stroke cases worldwide in 2016 (4, 120, 318/13, 676, 761), the total deaths (2,838,061 vs. 2,690,170) and disability-adjusted life-years lost (64.5 million vs. 51.9 million) from hemorrhagic stroke surpassed those of ischemic stroke [1]. A higher age at event onset and more severe functional outcomes for ICH patients than for ischemic stroke patients are common worldwide (Fig. 1) [2]. Nevertheless, the impact of acute therapy for ICH lags far behind that for acute ischemic stroke [3, 4]. An established therapeutic strategy for acute ICH analogous to reperfusion therapy for acute ischemic stroke has not been established. In the nationwide registry of the Japan Stroke Data Bank, functional outcomes improved for ischemic stroke patients over the past 20 years after age adjustment but did not improve for ICH patients [5]. The lack of an established strategy might be an essential reason for the difference.

Hypertension is a firmly established risk factor for ICH in the general population [6]. Elevated blood pressure (BP) is common after ICH and is reportedly associated with poor outcomes, presumably partly due to hematoma expansion [3, 4, 7, 8]. A theoretical risk for reducing cerebral blood flow surrounding hematomas by intensive BP lowering was also mentioned. Generally, there has been a consensus for many years that extremely high BP should be controlled during acute ICH. However, the BP goal had been set to be relatively high, with a systolic BP (SBP) of approximately 180 mmHg, due to the lack of scientific evidence for more intensive BP lowering. The recent revision to a lower goal of less than 140 mmHg required the publication of results in the mid-2010s from large, global, randomized, controlled trials, including the Second Intensive Blood Pressure Reduction in Acute Cerebral Haemorrhage Trial (INTERACT2) and the Antihypertensive Treatment of Acute Cerebral Hemorrhage (ATACH)−2 trial [9, 10].

Stroke Acute Management with Urgent Risk-factor Assessment and Improvement (SAMURAI) was the original name for the study group of multiple stroke registries funded by the Japanese government. The group completed a series of studies on three different themes: acute stroke thrombolysis [11, 12], anticoagulants for atrial fibrillation-associated stroke [13, 14], and BP management for acute ICH. The studies on the last theme were named the SAMURAI-ICH study. In the present review, changes in the strategy for BP lowering in acute ICH patients worldwide and in Japan over the last decade are presented based on the achievements of and evolved or derivative projects from the SAMURAI-ICH study.

Point of view **Clinical relevance**

Intensive blood pressure lowering in acute intracerebral hemorrhage using a strong antihypertensive agent such as intravenous nicardipine seems to improve functional outcome of patients, especially when hypertensive arteriopathy is the potential pathology of intracerebral hemorrhage.

**Future direction**

Appropriate blood pressure lowering from hyperacute to chronic stages coupled with novel developing therapeutic strategies will be essential for better recovery after intracerebral hemorrhage.

**Consideration for the Asian population**

Results from the SAMURAI-ICH and ATACH-2 were mainly derived from East Asian population; they are helpful for consideration of therapeutic recommendations of intracerebral hemorrhage in Asia.

## Trigger for the SAMURAI-ICH study

Of the etiological mechanisms for ICH, hypertensive arteriopathy seems to be relatively predominant in the Asian population, and cerebral amyloid angiopathy seems to be relatively predominant in the Western population [15]. Thus, intensive BP management would be more effective for preventing acute exacerbation after ICH onset in the Asian population. However, the Japanese Guidelines in the 2000s recommended acute BP lowering only when the BP was extremely high, i.e., an SBP >180 mmHg or a mean arterial pressure >130 mmHg, without indicating definite target BP levels [16], following the recommendation of the American Heart Association/American Stroke Association guidelines [17]. This recommendation was based on limited information without scientific evidence. To make matters worse, optimal intravenous antihypertensive agents for acute ICH patients were not established in Japan at that time. Of the representative intravenous antihypertensives recommended in Western countries [17], labetalol was not approved for commercial use, esmolol was used only as an antiarrhythmic drug, and the administration of nicardipine for hyperacute ICH patients was limited by the following description on the official label without scientific evidence: “nicardipine is contraindicated for (1) ICH patients with a suspicion of ongoing intracranial bleeding not to enhance bleeding and for (2) acute stroke patients with elevated intracranial pressure not to accelerate intracranial pressure elevation” [18]. One of the few remaining alternatives was diltiazem, although it often causes bradycardia or atrioventricular block. However, in our nationwide web survey in 2008, 57% of the respondents chose nicardipine as the first choice agent despite the official limitation [18]. To resolve the divergence between the official recommendation and actual clinical practice, the safety of nicardipine for Japanese ICH patients needs to be ascertained.

We had another reason for planning a clinical study on nicardipine use for acute ICH patients. In 2007, Dr. Hisatomi Arima (currently a Professor at the Department of Preventive Medicine and Public Health, Fukuoka University), a core member of the INTERACT trial, gave us an invitation from Sydney to join the INTERACT2 trial together with several Japanese sites that would soon be started. However, we had no experience and little capacity to direct multiple sites participating in international trials at that time. The refusal of this invitation was regrettable for us. The following year, Professor Yuko Palesch (Department of Public Health Sciences, Medical University of South Carolina), the coprincipal investigator of the ATACH-2 trial, visited our workplace, the National Cerebral and Cardiovascular Center, Suita, and invited us to join the trial. We did not want to miss this second chance. Since intravenous nicardipine was the only trial drug for the ATACH-2 trial, the official limitation of its use in Japan would need to be removed for trial participation.

We formed the SAMURAI study group with ten stroke centers in 2008 (Table 1). Acute BP lowering for ICH patients met our goal of “acute stroke management with an urgent risk factor assessment and improvement.” We immediately developed a study protocol.

**Table 1:** Participating institutions in SAMURAI-ICH and ATACH-2 from Japan

SAMURAI	ATACH-2	Institution	Site principal investigators
○	○	National Cerebral and Cardiovascular Center	Kazunori Toyoda, Kazuyuki Nagatsuka
○	○	Kobe City Medical Center General Hospital	Hiroshi Yamagami, Nobuyuki Sakai
○	○	Nakamura Memorial Hospital	Jyoji Nakagawara, Kenji Kamiyama
○	○	NHO Nagoya Medical Center	Satoshi Okuda
○	○	NHO Kyushu Medical Center	Yasushi Okada
○	○	Kohnan Hospital	Eisuke Furui, Ryo Itabashi
○	○	Kyorin University Hospital	Yoshiaki Shiokawa, Kazutoshi Nishiyama
○	○	St. Marianna University Hospital	Yasuhiro Hasegawa, Hisanao Akiyma
○	○	Kawasaki Medical School Hospital	Kazumi Kimura, Yoshiki Yagita
○		Jichi Medical University School of Medicine	Kazuomi Kario, Michito Namekawa
	**○**	Toranomon Hospital	Takayuki Hara
	**○**	Gifu University Hospital	Toru Iwama
	**○**	Saiseikai Central Hospital	Haruhiko Hoshino
	**○**	St. Marianna University Toyoko Hospital	Toshihiro Ueda
	**○**	Keio University Hospital	Yoshiaki Itoh, Takato Abe, Shinichi Takahashi

*NHO* National Hospital Organization

## Messages from the SAMURAI-ICH study

**Fig. 2:**
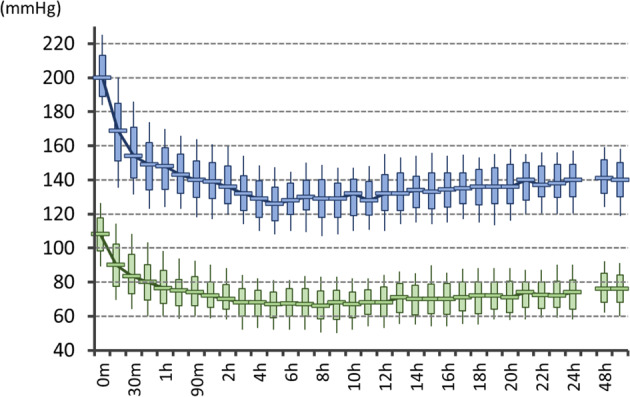
Trends in systolic and diastolic blood pressure levels during the 24-h administration of nicardipine: The SAMURAI-ICH study. Re-edited from Ref [19]. The boxes represent the interquartile ranges, the lines across the boxes indicate the median values, and the whiskers represent the 10th percentile and 90th percentile values

In the nationwide survey described above, 82% of the respondents chose the SBP goal to be ≤160 mmHg [18]. Thus, we planned a prospective, observational study, the SAMURAI-ICH study, to elucidate the safety and feasibility of the major choices from the survey of acute ICH patients to maintain SBP levels between 120 and 160 mmHg for 24 h using intravenous nicardipine [19]. We enrolled 211 patients (81 women, aged 65.6 ± 12.0 years, baseline SBP 201.8 ± 15.7 mmHg) with acute supratentorial ICH from July 2009 through June 2011. Using the strict titration method as used in the ATACH-2 trial, patients’ SBP levels were lowered to the target range in a median of 30 minutes [interquartile range (IQR) 15–45 minutes] with the proportion of time in the target SBP range over 24 h to be 77.6% (IQR 75.3–79.9%, Fig. 2). Neurological deterioration corresponding to a decrease of ≥2 points on the baseline Glasgow Coma Scale score or an increase of ≥4 points on the baseline National Institutes of Health Stroke Scale (NIHSS) score 72 h after the initiation of treatment was identified in 8.1% [95% confidence interval (CI) 5.1–12.5%] of the patients; serious adverse events requiring nicardipine be stopped within 24 h was reported in 0.9% (0.3–3.4%) of the patients; hematoma expansion >33% from baseline to 24 h was reported in 17.1% (12.6–22.7%) of the patients; and a poor outcome corresponding to modified Rankin Scale (mRS) scores of 4 to 6 at 3 months was reported in 41.2% (34.8–48.0%) of the patients. The rates were less than the upper limit of the 90% CI for the predicted proportion based on the weighted average of previous studies (25.9%, 8.9%, 28.3% and 67.9%, respectively) and some were even equal to or less than the lower limit (15.2%, 1.8%, 17.1%, and 54.5%, respectively). We concluded that SBP lowering to ≤160 mmHg using intravenous nicardipine appeared to be well tolerated and feasible for Japanese acute ICH patients.

While proceeding with patient registration for the SAMURAI-ICH study, we submitted the interim results of the study together with the results of the nationwide survey described above as references in a petition by the Japan Stroke Society to the Ministry of Health, Labour, and Welfare of Japan to revise the rule regarding the contraindication for ICH on the official label of intravenous nicardipine. The rule was finally abolished in 2011; it enabled us to join the ATACH-2 trial.

**Fig. 3:**
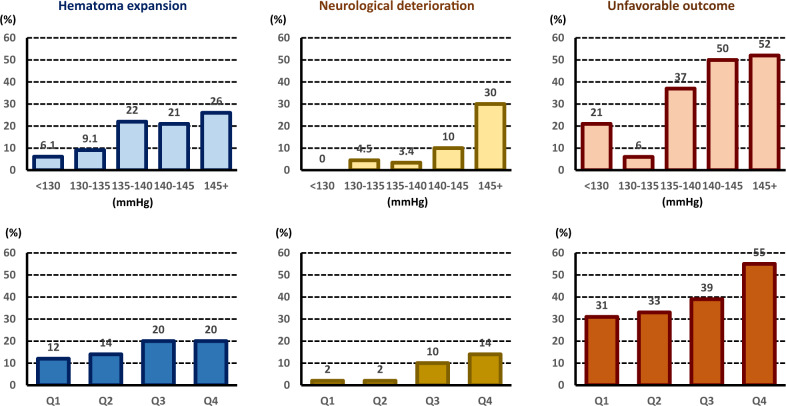
Correlations between the rates of clinical outcomes and mean systolic blood pressure levels (5-mmHg-interval categories, top panels) or their successive variation (quartiles, bottom panels) during the 24-h administration of nicardipine: The SAMURAI-ICH study. Re-edited from Ref [20, 22]

The SAMURAI Investigators published several substudies using the dataset; the themes included the association of clinical outcomes with mean SBP and its variability during the initial 24 h [20–23], the timing of SBP lowering to the target level [24], the total dosing of nicardipine [25], kidney function [26], and blood glucose levels (Table 2) [27]. Another unique theme was conjugate eye deviation during acute ICH [28]. The top panels of Fig. 3 show the correlations between the rates of clinical outcomes and mean achieved SBP levels during the 24-h administration of nicardipine [20]. All of the rates of hematoma expansion within 24 h, neurological deterioration at 72 h, and unfavorable outcomes at 3 months increased as the mean SBP levels increased. The mean SBP was independently associated with hematoma expansion (odds ratio 1.86, 95% CI 1.09–3.16 per 10 mmHg), neurological deterioration (4.45, 2.03–9.74), and unfavorable outcomes (2.03, 1.24–3.33) after adjusting for the known predictors. The bottom panels of Fig. 3 show the correlations between the rates of clinical outcomes and successive variations in SBP during the initial 24 h [22]. The rates of neurological deterioration and poor outcomes increased as the successive variations increased. Successive variation in SBP, a representative indicator of variability, was independently associated with neurological deterioration (odds ratio 2.37, 95% CI 1.32–4.83 per quartile category) and unfavorable outcomes (1.42, 1.04–1.97) after adjusting for the known predictors. Early intensive and stable SBP lowering after onset seemed to improve the clinical outcomes of ICH patients.

**Table 2:** Main findings of substudies from SAMURAI-ICH

Reference	Theme	Finding
#^20^ (Sakamoto 2013)	Mean systolic blood pressure (SBP) during 24 h	High achieved SBP after standardized antihypertensive therapy in acute supratentorial intracerebral hemorrhage (ICH) was independently associated with poor clinical outcomes.
#^21^ (Kobayashi 2014)	Three time periods of SBP during 24 h	Higher SBP levels during 0–8 h and during 8–16 h were independently associated with neurological deterioration, and higher SBP levels during 8–16 h and during 16–24 h were independently associated with poor outcomes.
#^22^ (Tanaka 2014)	SBP variability	SBP variabilities (standard deviation, successive variation) during the initial 24 h were independently associated with neurological deterioration and poor outcomes.
#^23^ (Sakamoto 2015)	Relative SBP reduction	Insufficient relative SBP reduction was independently associated with poor clinical outcomes.
#^24^ (Yamaguchi 2018)	Timing of SBP lowering	Early achievement of target SBP < 160 mmHg was associated with a lower risk of hematoma expansion.
#^25^ (Koga 2014)	Total dose of nicardipine	Nicardipine dose needed for 24-h SBP lowering was roughly predictable by sex, age, body weight, and initial SBP. The maximum dose was associated with neurological deterioration.
#^26^ (Miyagi 2015)	Kidney function	Initial estimated glomerular filtration rate <60 mL/minute/m^2^ was associated with poor clinical outcomes.
#^27^ (Koga 2015)	Blood glucose	High blood glucose levels at 24 and 72 h were independently associated with poor clinical outcomes.
#^28^ (Sato 2012)	Conjugate eye deviation	The persistence of conjugate eye deviation was a significant predictor of death or dependency after acute supratentorial ICH even after adjusting for initial severity and hematoma volume.

## Moving on to the ATACH-2 trial

**Fig. 4:**
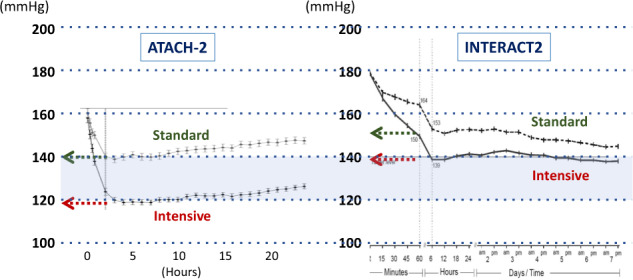
Trends of mean hourly systolic blood pressure in the ATACH-2 and INTERACT2 trials. Re-edited from Ref [9, 10]. Achieved systolic blood pressure levels in the standard treatment group of the ATACH-2 trial and the intensive treatment group of the INTERACT2 trial were similar (~140 mmHg) and that in the intensive treatment group of the ATACH-2 trial was lowered to ~120 mmHg

Encouraged by the success of the SAMURAI-ICH study, 14 Japanese sites participated in the ATACH-2 trial (Table 1). The ATACH-2 trial was a randomized, multicenter, two-group, open-label trial to determine the relative efficacy of intensive (110–139 mmHg) versus standard (140–179 mmHg) SBP lowering with intravenous nicardipine using a strictly defined titration method that was initiated within 4.5 h after symptom onset and continued for the next 24 h in patients with spontaneous supratentorial ICH [10]. Of the 1,000 participants, 288 were enrolled from Japan, 246 from other Asian countries (China, Taiwan, and South Korea), and the remaining 466 from the United States and Germany. The trial did not show a benefit in reducing the rate of the primary outcome of death or disability, defined as an mRS score of 4–6, between the two treatment groups (relative risk with intensive treatment 1.04, 95% CI 0.85–1.27). The result was somewhat different in the INTERACT2 trial, which overlapped in time with the ATACH-2 trial, showing possibly better functional outcomes for acute ICH patients with early intensive SBP lowering (<140 mmHg) than for patients with standard lowering (<180 mmHg) with the use of any antihypertensive agents of a physician’s choosing (odds ratio 0.87, 95% CI 0.75–1.01) [9]. The differences in the results seemed to be partly caused by the considerable variance of achieved SBP levels between the two trials, as shown in Fig. 4, although the target SBP levels of each treatment group were the same. The optimal SBP goal for acute ICH patients might be between the mean achieved SBPs of the intensive treatment groups of both trials (between 120 and 140 mmHg).

Japanese researchers contributed to the ATACH-2 trial not only by patient recruitment but also by subanalyses using the ATACH-2 dataset. The themes of the subanalyses included sex differences [29], regional differences (Asia versus non-Asia) [30], late neurological deterioration [31], kidney function [32], heart rate [33], and the impacts of achieved SBP levels on clinical outcomes [34].

**Fig. 5:**
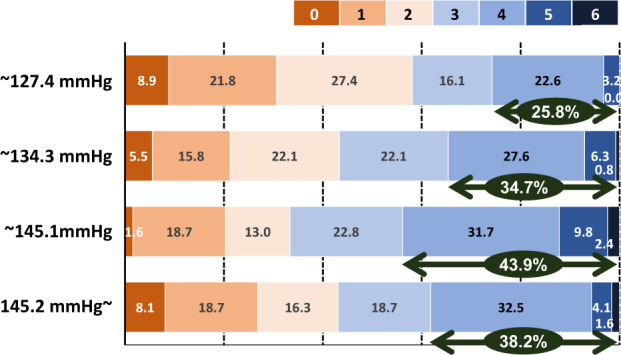
Modified Rankin Scale scores at 90 days for Japanese patients with acute intracerebral hemorrhage divided into quartile groups by mean systolic blood pressure levels during the 24-h administration of nicardipine: The pooled analysis. Re-edited from Ref [36]. Arrows indicate the percentage with modified Rankin Scale scores of 4–6

Finally, the results from a systematic review and individual participant data analysis using the combined database from the SAMURAI-ICH study, ATACH-2 trial, and ATACH-1 trial [35], a small pilot trial for the ATACH-2 trial, are presented [36]. Prospective studies before October 1, 2020, were identified in PubMed; studies involving hyperacute ICH adult patients treated with intravenous nicardipine whose outcomes was assessed using the mRS score were eligible (PROSPERO: CRD42020213857), and the above three studies met the eligibility criteria. For the 1,265 patients enrolled (484 women, aged 62.6 ± 13.0 years, baseline SBP 206.1 ± 21.0 mmHg), the mean hourly SBP during the initial 24 h was positively associated with an mRS score of 4–6 (adjusted odds ratio 1.12, 95% CI 1.00–1.26 per 10 mmHg) and hematoma expansion within 24 h (1.16, 1.02–1.32). A total of 499 patients (183 women, aged 64.9 ± 11.8 years, baseline SBP 203.5 ± 18.3 mmHg) from Japan were registered in this pooled analysis. For Japanese patients, the mean hourly SBP was more strongly associated with an mRS score of 4–6 (adjusted odds ratio 1.26, 95% CI 1.04–1.53) and hematoma expansion (1.47, 1.17–1.85) than for the overall participants (Table 3). When the mRS score was compared among the quartiles by the mean hourly SBP during the initial 24 h, the distribution shifted to higher scores as the SBP became higher in the first three quartiles (Fig. 5).

**Table 3:** Associations of mean hourly systolic blood pressure between 1 and 24 h with outcomes

	Overall subjects			Japanese subjects		
	Odds ratio^a^	95% CI^a^	*P*	Odds ratio^a^	95% CI^a^	*P*
Death or disability	(*n* = 1,211)		(*n* = 497)			
	1.12	1.00–1.26	0.0470	1.26	1.04–1.53	0.0198
Hematoma expansion	(*n* = 1,194)		(*n* = 486)			
	1.16	1.02–1.32	0.0207	1.47	1.17–1.85	0.0010

Data on overall subjects are derived from Ref [36]. Data on Japanese subjects are newly analyzed for this review article.

Adjusted for sex, age, study group, race, baseline National Institutes of Health Stroke Scale, baseline hematoma volume, hematoma site, and onset-to-randomization time

^a^per 10 mmHg

As described above, hypertensive arteriopathy seems to be predominant in Japanese patients [15]. Thus, intensive SBP lowering might show a strong preventive effect in a Japanese cohort. It should also be noted that acute kidney injury and renal adverse events were known complications in the ATACH-2 trial, especially when the SBP was lower [29, 34, 37]. Attention to changes in kidney function is accordingly indispensable when intensively lowering SBP.

The 2010s was a decade for changing the strategy of acute BP lowering after ICH onset. In Japan, we revised the official label of intravenous nicardipine for its appropriate use in hyperacute ICH patients, participated in the memorable and global BP-lowering therapy trial, and clarified details regarding the therapy using international datasets that involved many Japanese patients throughout the decade. The SAMURAI-ICH study helped a series of these activities. In the present decade, new pharmacotherapeutic strategies such as emergent hemostasis will be developed [4, 38]. Appropriate BP lowering from hyperacute to chronic stages, coupled with such novel strategies, will be essential for the better recovery of patients after ICH.
